# Efficiency comparison between tracking and optimally fixed flat solar collectors

**DOI:** 10.1038/s41598-023-39892-y

**Published:** 2023-08-05

**Authors:** Amir Aghamohammadi, M. Ebrahim Foulaadvand

**Affiliations:** 1 Faculty of Physics, Alzahra University, Tehran, Iran; 2 Department of Physics, Faculty of Science, University of Zanjan, Zanjan, 45371-38791 Iran

**Keywords:** Physics, Astronomy and astrophysics

## Abstract

We investigate the optimal orientation for a fixed flat plate solar collector using the clear sky model. The ground reflection component of irradiation that hits the collector’s surface is ignored due to its relatively small magnitude when compared to the direct beam and sky diffusive components. Analytical calculations demonstrate that regardless of the collector’s latitude, the most effective azimuthal angle, $\gamma ^*$, is 0, which generally corresponds to a North–South direction. However, the optimal tilt angle, $\beta ^*$, is dependent on both the Day of  Year (DoY) and the collector’s local latitude. For latitudes typical of mid-altitude climate zones, we can calculate the optimal tilt angle and the maximum energy that the collector can harvest during each DoY. We compare the maximum daily received energy—which is the sum of the direct beam and sky diffusive energies—associated with this optimal orientation to their corresponding values when the flat plate tracks the Sun. The relative increase in total energy due to Sun tracking depends critically on the DoY, with a minimum value of about $17\%$ in early winter and a maximum value of $40\%$ over a large interval.

## Introduction

Devices such as solar collectors, panels, and concentrators are designed to harvest energy from the Sun’s radiation^1–7^. Maximizing their performance and efficiency is crucial, and the most effective way to achieve this is by orienting the collector along the Sun’s beam, known as the Direction Normal Irradiance (DNI). However, this requires a tracking system, as the Sun’s apparent position in the sky changes throughout the day. While tracking systems can significantly improve efficiency, they can also be expensive and require additional energy for operation^8^. Moreover, their operation and maintenance are also costly. To reduce these costs, it is desirable to place solar collectors at a fixed but optimal orientation and periodically adjust this orientation as needed. However, finding the optimal orientation is not an easy task and depends on several extrinsic factors, including climatological and meteorological conditions^9,10^. Typically, the optimal orientation of a solar collector is determined empirically on a daily, monthly, quarterly, or annual basis. There are some factors which may affect the amount of received irradiation by a solar collector. The received irradiation may depend on the geometry and the shape of solar collector. Moreover, it depends on the latitude of the location, the day of the year, and also the climate. As a result, determining the optimal orientation can be a complex and location-dependent process. Many solar collectors have a flat surface, such as flat plate collectors and PV panels, while others have a concave curvature, such as solar dishes or parabolic troughs. However, in the case of curved collectors, the effective surface area that is exposed to the Sun (aperture) is flat. The orientation of a flat-aperture collector can be specified by two angles of tilt, $\beta$, and azimuth, $\gamma$. In recent years several research groups have been perusing the optimization of solar collector orientation for different locations around the world. Different techniques, including genetic algorithms and simulated annealing, have been used^11–38^. For a detailed review see^39^. Most articles that address the problem of optimal orientation for flat surface collectors have done so on a local and non-universal geographic scale. As a rule of thumb, it is suggested that in the Northern (Southern) hemisphere, the optimal orientation is south (north)-facing, and that the optimal annual tilt angle should be the same as the local latitude. However, other papers have proposed a wider range for the optimal tilt angle^11,15,16^. Unfortunately, many of these investigations suffer from a lack of a comprehensive and rigorous mathematical approach. In this paper, we aim to address the problem of optimizing fixed-orientation solar collectors using a rigorous mathematical framework. It may seem intuitive that the collector’s optimal orientation is perpendicular to the direction of the sun’s rays at solar noon, as sunlight shines almost directly overhead during that time. However, as we will see, considering the contribution of direct irradiation energy throughout the day, including radiation in the early morning and afternoon, the optimal tilt angle deviates from this conjecture. As we will see, it crucially depends both on the latitude and the day of the year. The total solar irradiation received on the ground consists of three main components: direct beam, sky diffusive, and ground reflection. While the contribution of ground reflection is negligible, the contribution of sky diffusive radiation is significant. In this study, we focus on the direct beam and sky diffusive components, and ignore the ground reflection. Specifically, we calculate the energy contributions due to the direct beam and sky diffusive radiation separately, with the latter being investigated using an isotropic approximation. In this paper we do not consider the impact of irradiation incidence angle on the characteristics of solar energy conversion. As an example, the efficiency of solar PV panels are affected by the angle at which the solar rays hit the panel^40–42^ or in solar concentrators, diffusive irradiation cannot be harvested. This important and challenging problem requires further investigation. Moreover technology-dependent efficiency could be interesting for future considerations. Here, our primary focus is on the overall received irradiation energy of a flat collector rather than delving into energy conversion and panel efficiency details. This paper is organized as follows: in “Some astronomy” section, some mathematical astronomy prerequisites are presented; in “Formulation and methodology: optimal orientation of a flat plate receiver” section, we discuss the optimal orientation of a flat solar collector and provide an analytical solution for the optimal angles; in “Comparison with a tracking flat plate” section, we compare the total energy harvested by a fixed flat plate and a tracking one, and present our findings; “Comparison to existing results in the literature” section is dedicated to comparing our results with similar existing findings in the literature. And finally we conclude the paper with some final remarks.

## Some astronomy

To describe the Sun-Earth geometry, one needs a system of coordinates. There are two main pictures: heliocentric and geocentric. In the heliocentric picture, the Sun (at the origin) is placed at one of the foci of an ellipse with a small eccentricity around 0.0167. The Earth orbits around the Sun in an elliptical path on a plane called the *ecliptic*. The plane perpendicular to the Earth axis (which connects the North pole to the South one at the Earth’s center) is the Earth equatorial plane. It is inclined to the ecliptic plane by an obliquity angle $23.45^\circ$. In the geocentric system of coordinates, the Earth is at the origin. There are two choices for the $x-y$ plane: equatorial and horizontal which will be briefly explained.

### Horizontal picture

**Figure 1:**
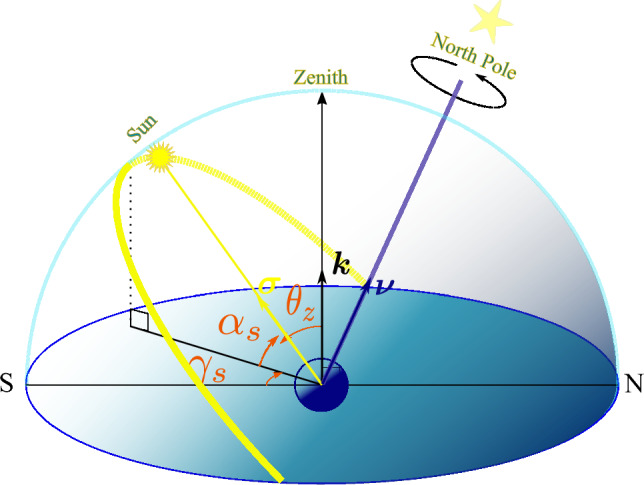
Sun’s angular position is given by two angles elevation $\alpha _s$ and azimuth $\gamma _s$ with the observer's horizon as the fundamental ($x-y$) plane.

In the horizontal perspective, the system of coordinates is established with the observer as the origin, the observer’s horizon as the fundamental $x-y$ plane, and the *z*-axis along the zenith, i.e., the observer’s overhead direction towards the sky. The Sun’s angular position is described by two angles of elevation, or altitude, $\alpha _s$, and azimuth $\gamma _s$. Figure 1 provides an illustration. The zenith angle, $\theta _z$, and the solar altitude angle, $\alpha _s$, are complementary. So, $\theta _z = \frac{\pi }{2} - \alpha _s$. This angle represents the direct beam irradiation angle. The unit vector ${\varvec{\sigma }}$ specifies the line connecting the observer to the Sun, while the unit vector ${\varvec{\nu }}$ specifies the North pole direction, i.e., the line connecting the Earth’s center to its North pole.

### Equatorial picture

**Figure 2:**
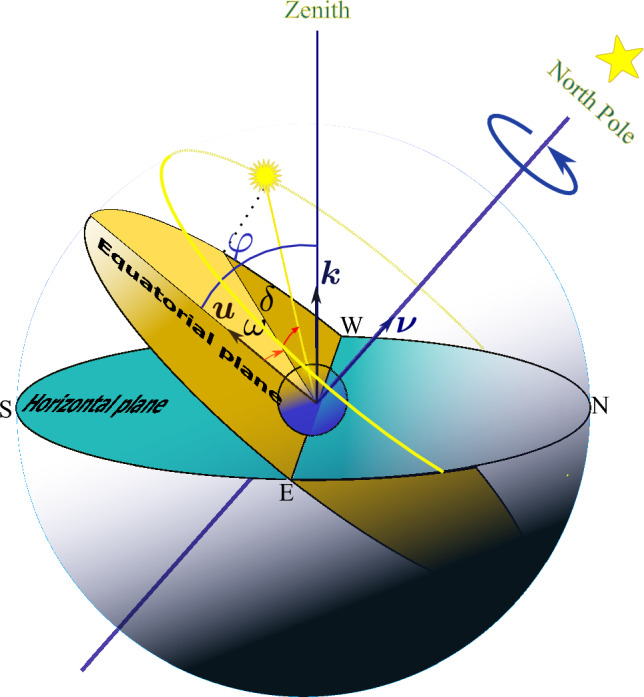
Equatorial geocentric system of coordinates: Sun angular position is characterised by two angles declination $\delta$ and hour $\omega$.

In the equatorial perspective, the origin is placed at the Earth’s center. In this picture the fundamental plane is the equatorial plane that passes through the terrestrial equator. The Sun’s angular position is specified by two angles: declination, $\delta$, and hour angle, $\omega$. The angle between the equatorial plane and the line connecting the Sun to the origin (Earth) is the declination angle, shown by $\delta$. Alternatively, the declination angle, $\delta$, is the Sun’s altitude with respect to the equatorial plane. To specify the hour angle, $\omega$, we first need to define a local meridian. Earth is at the origin of the Celestial sphere. The local celestial meridian is the circle on the celestial sphere. It is perpendicular to both the horizontal plane and the equatorial plane. The zenith, nadir, North Celestial Pole, and South Celestial Pole are located on the celestial meridian. The hour angle, $\omega$, is defined as follows: first, project the line connecting the origin to the Sun onto the equatorial plane. The hour angle $\omega$ is the angle between these two lines: the Sun projection on the equatorial plane and the line connecting the origin and the intersection point of the local meridian with the equatorial plane. Figure 2 provides an illustration.

Angles $\gamma _s$ and $\alpha _s$ are related to the declination angle $\delta$, hour angle $\omega$ and latitude $-\frac{\pi }{2}<\varphi <+\frac{\pi }{2}$ according to the following trigonometric formulas:

1 $$\begin{aligned} {\varvec{\sigma }}= & {} {\varvec{\nu }}\sin \delta + {\varvec{e}}_{\textrm{w}}\cos \delta \sin \omega +{\varvec{u}}\cos \delta \cos \omega , \end{aligned}$$

2 $$\begin{aligned} {\varvec{\sigma }}= & {} {\varvec{k}}\sin \alpha _s+ {\varvec{e}}_{\textrm{s}}\cos \alpha _s \cos \gamma _s+{\varvec{e}}_{\textrm{w}}\cos \alpha _s \sin \gamma _s, \end{aligned}$$

where we have used of ${\varvec{u}}= {\varvec{e}}_{\textrm{s}} \sin \varphi +{\varvec{k}}\cos \varphi$. Here ${\varvec{e}}_{\textrm{s}}$ and ${\varvec{e}}_{\textrm{w}}$ are unit vectors toward South and West. Using the above relations and ${\varvec{\nu }}= -{\varvec{e}}_{\textrm{s}} \cos \varphi +{\varvec{k}}\sin \varphi$, one arrives at:

3 $$\begin{aligned} {\varvec{\sigma }}\cdot {\varvec{k}}&=\cos \theta _z= \sin \alpha _s=\cos \varphi \cos \delta \cos \omega +\sin \varphi \sin \delta , \end{aligned}$$

4 $$\begin{aligned} {\varvec{\sigma }}\cdot {\varvec{e}}_{\textrm{s}}&= {\varvec{e}}_{\textrm{s}}\cdot {\varvec{\nu }}\sin \delta + {\varvec{e}}_{\textrm{s}}\cdot {\varvec{u}}\cos \delta \cos \omega = \cos \alpha _s \cos \gamma _s,\nonumber \\ {}&=-\sin \delta \cos \varphi +\sin \varphi \cos \delta \cos \omega =\cos \alpha _s \cos \gamma _s \end{aligned}$$

5 $$\begin{aligned} {\varvec{\sigma }}\cdot {\varvec{e}}_{\textrm{w}}&= \cos \delta \sin \omega = \cos \alpha _s \sin \gamma _s, \end{aligned}$$

Now, let’s turn to the objective of finding the optimal static orientation of solar collectors to harvest the maximum amount of solar irradiation.

## Formulation and methodology: optimal orientation of a flat plate receiver

It’s clear that the maximum amount of energy can be received on the collector’s aperture if it tracks the Sun’s movement, but as we have noted, such a tracking mechanism can be expensive. When we refer to the optimal configuration of a solar collector, we are talking about specifying the collector’s fixed orientation, which includes the two angles of tilt and azimuth. The objective is to determine the orientation that maximizes the total amount of irradiation that impinges on the collector’s surface during a given time period. The time period that is spent to measure the irradiation time may vary from a single day to a month, a season or even a year depending on our desired application. For the purpose of determining the optimal orientation for a solar collector, we will consider the shortest period, which is one day. With daily data for the optimal orientation, the procedure for longer periods can be easily extrapolated. For our purposes, we will focus on the simplest collector geometry, which is a flat plate collector with a unit area. The flat plate collector is located at a local latitude of $\varphi$, and its orientation is determined by the direction of its unit normal vector, ${\varvec{n}}$. In the spherical coordinates system of an observer, where the $x-y$ plane is the local horizontal plane and the ${\varvec{k}}$ direction is aligned with the zenith, this unit normal vector can be specified by two angles: the tilt angle, $\beta$, and the azimuthal angle, $\gamma$. The unit vector can be decomposed as follows:

6 $$\begin{aligned} {\varvec{n}}= {\varvec{k}}\cos \beta + {\varvec{e}}_{\textrm{s}}\sin \beta \cos \gamma +{\varvec{e}}_{\textrm{w}}\sin \beta \sin \gamma . \end{aligned}$$

**Figure 3:**
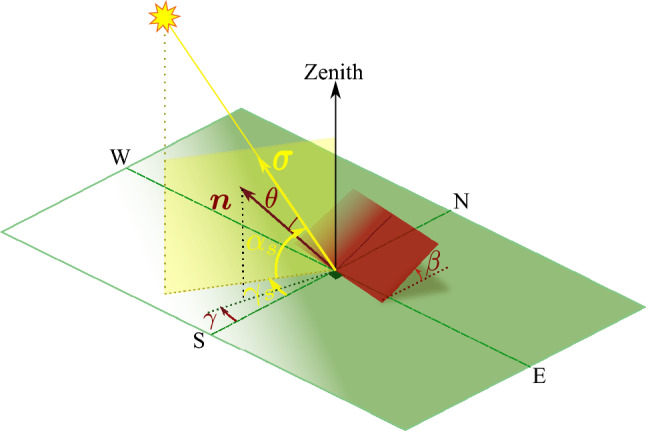
Specification of a flat plate orientation by two angles: tilt angle, $\beta$, and azimuth angle, $\gamma$, in the local observer’s system of coordinates, specified by the horizon plane and the zenith.

Please refer to Fig. 3 for an illustration of the flat plate collector’s orientation. Note that the azimuthal angle $\gamma$ is measured positively from the South towards the West.

If we take $-\dfrac{\pi }{2} \le \beta \le \dfrac{\pi }{2}$, then positive (negative) values for $\beta$ indicate that the panel is oriented towards the South (North). The unit vector ${\varvec{n}}$ can be expanded in terms of ${\varvec{\nu }}$ and the two other perpendicular unit vectors of the equatorial plane in a similar manner. The incidence angle $\theta$ is defined as the angle between the normal to the plate, ${\varvec{n}}$, and the direction of the Sun, which is the line connecting the observer to the Sun, denoted by ${\varvec{\sigma }}$. In the equatorial plane $\varvec{n}$ can be decomposed as:

7 $$\begin{aligned} \varvec{n}=\varvec{\nu } \cos \eta +\varvec{u} \sin \eta \cos \zeta +\varvec{e}_{\textrm{w}} \sin \eta \sin \zeta . \end{aligned}$$

**Figure 4:**
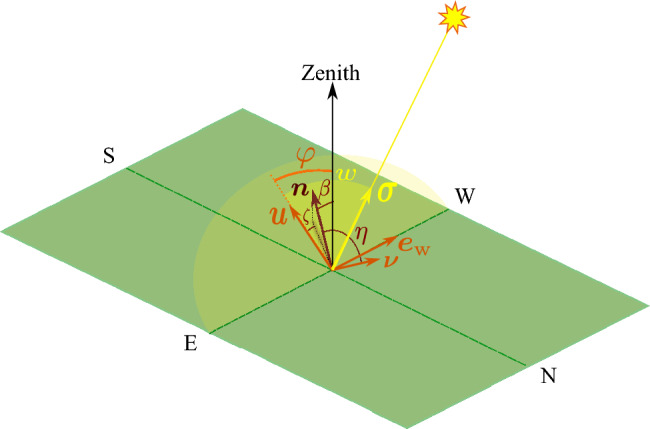
Specification of a flat plate orientation, ${\varvec{n}}$, by two angles: tilt angle, $\eta$, and azimuth angle, $\zeta$, defined with respect to the equatorial plane and the vector ${\varvec{\nu }}$. The equatorial plane is indicated by a pale yellow semi-circle.

The angle between ${\varvec{n}}$ and ${\varvec{\nu }}$ is denoted by $\eta$, while the angle between the projection of ${\varvec{n}}$ onto the equatorial plane and the unit vector ${\varvec{u}}$ within that plane is denoted by $\zeta$. Please refer to Fig. 4 for an illustration of these angles.

By utilizing Eqs. (1), (2), (6), and (7), we arrive at

8 $$\begin{aligned} \cos \theta&={\varvec{\sigma }}\cdot {\varvec{n}}\nonumber \\ \cos \theta&=\sin \delta \sin \varphi \cos \beta -\sin \delta \cos \varphi \sin \beta \cos \gamma +\cos \delta \cos \varphi \cos \beta \cos \omega \nonumber \\&\quad +\cos \delta \sin \varphi \sin \beta \cos \gamma \cos \omega +\cos \delta \sin \beta \sin \gamma \sin \omega . \end{aligned}$$

9 $$\begin{aligned} \cos \theta&=\sin \delta \cos \eta + \cos \delta \sin \eta \cos ( \omega - \zeta ). \end{aligned}$$

Equations (8) and (9) provide $\cos \theta$ in terms of the Sun’s positional angles ($\delta$ and $\omega$) in the geocentric equatorial system of coordinates, the observer’s latitude $\varphi$, and the flat plate’s orientation angles, $\beta$ and $\gamma$, or equivalently, $\eta$ and $\zeta$. If we consider the special case of $\gamma = 0$ (or equivalently, $\zeta = 0$), the calculations become simpler. The vector $\varvec{n}$ is in the plane constructed by $\varvec{u}$ and $\varvec{\nu }$ ($\varvec{k}$ and $\varvec{e}_s$). Therefore, we can express ${\varvec{n}}$ as follows:

10 $$\begin{aligned} \varvec{n}= & {} \varvec{\nu } \cos \eta +\varvec{u}\sin \eta , \end{aligned}$$

11 $$\begin{aligned}= & {} \varvec{k} \cos \beta +\varvec{e}_s\sin \beta . \end{aligned}$$

Our objective is to determine the total amount of radiation energy that the flat plate receives on the *n*th day of the year, where $n=1$ corresponds to the first of January in the Julian calendar. The total instantaneous irradiation, *G*, that impinges on a surface is composed of three components: beam direct radiation, $G_b$, sky diffuse radiation, $G_d$, and ground diffuse reflection, $G_r$. The beam direct radiation, $G_b$, typically provides the largest contribution to the total irradiation. Since the ground reflection component is small compared to $G_b$ and $G_d$, it will be neglected in this paper. To begin, we will focus on the energy, $E_b$, received by the flat collector due to the beam direct irradiation, $G_b$.

### Contribution of beam direct radiation to received energy

The total direct irradiation energy during a day (from sunrise to sunset) received on a flat plate of unit area is:

12 $$\begin{aligned} E_{\textrm{b}}(n,\varphi )=\int\limits _{t_{\textrm{r}}}^{t_{\textrm{s}}}\textrm{d}t\, G_{\textrm{bn}}(n,t)\cos \theta \, \Theta [\cos \theta ]. \end{aligned}$$

where the variables $t_{\textrm{r}}$ and $t_{\textrm{s}}$ represent the local times of sunrise and sunset, respectively. $G_{\textrm{bn}}$ is the magnitude of the direct normal beam, and ${\varvec{G}}_{\textrm{bn}}=G_{\textrm{bn}}(-{\varvec{\sigma }})$ represents the direct normal beam vector. It should be noted that $t_{\textrm{r}}$ and $t_{\textrm{s}}$ are functions of *n* and $\varphi$, but for brevity, we do not explicitly write them. The variable $\Theta$ is the Heaviside step function, which ensures that the surface receives irradiation when the following condition holds

13 $$\begin{aligned} {\varvec{G}}_{\textrm{bn}}\cdot ({-\varvec{n}})=G_{\textrm{bn}}\, {\varvec{\sigma }}\cdot {\varvec{n}}=G_{\textrm{bn}}\cos \theta >0. \end{aligned}$$

To evaluate the integral in Eq. (12), we need to specify the dependence of the Sun’s geocentric coordinate angles $\delta$ and $\omega$ on the day of the year (DoY) and local time. The declination angle has a weak dependence on local time, so we neglect it in this paper unless stated otherwise. The dependence of the declination angle $\delta$ (in radians) on the day of the year (n) is given by^43,44^:

14 $$\begin{aligned} \delta =\dfrac{23.45^\circ \pi }{180}\sin \left( \frac{2\pi (284+n)}{365}\right) . \end{aligned}$$

In contrast to the declination angle, the hour angle $\omega$ is solely a function of time, specifically the hour of the day (HoD). We use *solar time*, which is based on the apparent angular motion of the Sun across the sky. Since the Earth rotates by $15^\circ$ per hour around its axis, the relationship between solar time and hour angle in terms of degrees (radians) per second can be expressed as:

15 $$\begin{aligned} \Omega =\frac{\Delta \omega }{\Delta t}=\frac{15^\circ }{h}=\frac{1^\circ }{4\,{\textrm{min}}}=\frac{\mathrm{0.0041}^{\circ }}{\textrm{sec}}=7.15\times 10^{-5}~{\textrm{rad}}/{\textrm{s}}. \end{aligned}$$

Defining $\omega =0$ at solar noon ($h=12:00\, \textrm{h}$) the hour angle $\omega$ can be determined for any solar time hour. The last step would be to calculate the local times $t_{\textrm{r}}$ and $t_{\textrm{s}}$ (for a given DoY, *n*, and latitude $\varphi$) in terms of solar time $t_{\textrm{sol}}$. For this purpose, let us first write the equation relating local time (standard time) $t_{\textrm{std}}$ and solar time $t_{\textrm{sol}}$:

16 $$\begin{aligned} t_{\textrm{sol}}=(L_r-L_{\textrm{loc}})\left( 4\frac{\textrm{min}}{1^\circ }\right) +EoT+t_{\textrm{std}}. \end{aligned}$$

with ${\textrm{EoT}}$ (Equation of Time):

17 $$\begin{aligned} EoT&=180\cdot \frac{4}{\pi }\left[ 0.000075+0.001868\cos (d)-0.032077\sin (d)\right. \nonumber \\&\quad \left. -0.014615 \cos (2d)-0.0409\sin (2d)\right] {\textrm{min}}, \end{aligned}$$

$d=\dfrac{2\pi (n-1)}{365}$^44^, $L_{\textrm{loc}}$ is the local longitude, and $L_r=({\textrm{LCT}}-{\textrm{GMT}})\times 12.5^\circ /\textrm{hour}$ is the reference longitude. LCT is the local civil time and GMT is Greenwich Mean Time. For example, Tehran civil time is GMT$+3.5~{\textrm{hours}}$ hence for Tehran we have: $L_r=3.5\times 15^\circ =52.5^\circ$. Principally we change the integral variable from standard time *t* into solar time $t_{\textrm{sol}}$ in Eq. (12). However, we need a second change of variable from solar time $t_{\textrm{sol}}$ to hour angle variable $\omega$ in the integrand. It turns out:

18 $$\begin{aligned} E_{\textrm{b}}(n,\varphi )=\frac{1}{\Omega }\int \limits ^{\omega _{\textrm{s}}}_{\omega _{\textrm{r}}}G_{\textrm{bn}}(n,\omega )\cos \theta (n,\omega )\Theta [\cos \theta (n,\omega )]d\omega . \end{aligned}$$

where $$\omega _{\textrm{r}}$$ is the Sun’s hour angle at sunrise and $$\omega _{\textrm{s}}$$ is the Sun’s hour angle at sunset. These are obtained from (3) by setting $$\alpha _s=0$$. This equation ($$\cos \omega =-\tan \varphi \tan \delta$$) has two solutions $$\omega _{\textrm{r}}$$ and $$\omega _{\textrm{s}}=-\omega _{\textrm{r}}$$ correspondingly. The hour angle dependence of the direct normal beam, $$G_{\textrm{bn}}(n,\omega )$$, makes the integral difficult to evaluate. In the following subsection, we will briefly review this dependence. The solar energy that reaches the Earth is the electromagnetic energy emitted by the Sun which to a good extent can be approximated to be a black body with surface temperature $$5777~{\textrm{K}}$$. As the light travels a long distance between the Sun and Earth (average distance $$1.496\times 
10^{11}~{\textrm{m}}$$) this flux of energy can be assumed to reach the outer region of the Earth’s atmosphere in the form of a plane wave. This radiative flux, the energy per unit time received on a surface of unit area perpendicular to the propagation direction, is named solar constant and is denoted by $$G_{\textrm{sc}}$$. It can be easily verified that the solar constant has a value $$G_{\textrm{sc}}=1367 \dfrac{\textrm{W}}{\textrm{m}^2}$$^1,45^ at the mean Sun-Earth distance. Due to the Earth’s orbit eccentricity around the Sun, the solar irradiation outside the Earth’s atmosphere (extraterrestrial irradiation) $$G_{on}$$ has a dependence on DoY. It can be approximated in the following way:

19 $$\begin{aligned} G_{\textrm{on}}(n)=G_{\textrm{sc}}\left( 1+0.033\cos \left( \frac{2\pi n}{365}\right) \right) . \end{aligned}$$

In the subscript of $G_{\textrm{on}}$, note that $``\textrm{o}''$ refers to “outside” and $``\textrm{n}''$ refers to “normal”. Due to extinction processes such as Rayleigh or Mie scattering, or absorption, the amount of irradiance that reaches the Earth’s surface is less than the amount outside of the atmosphere. Taking into account all of these attenuation effects, the amount of irradiance at ground level, denoted by $G_{\textrm{bn}}$, can be approximated using the following formula:

20 $$\begin{aligned} G_{\textrm{bn}}(n,\omega )=G_{\textrm{on}}(n)\tau _b. \end{aligned}$$

Here, $\tau _b$ represents the effective atmospheric transmission coefficient of the direct beam. Various models, each with their own set of assumptions and parameters, have been proposed to estimated the amount of $\tau _b$. Each model has its own advantages and limitations. In this work our focus is on a clear sky condition where there is no cloud in the sky and the atmosphere over the studied location is free of pollutants. Under this assumption, a wide range of *clear sky* models exists in the literature. For simplicity, we assume a clear sky model proposed in^46^, in which the sky is cloudless, clear (visible up to 23 km), and pollution-free. According to Hottel’s model, the effective atmospheric optical transmission coefficient $\tau _b$ is:

21 $$\begin{aligned} \tau _b=a_0+a_1\exp \left( -\frac{k}{\cos \theta _z}\right) . \end{aligned}$$

The constants $a_0,a_1$ and *k* are altitude and climate type dependent. See chapter two of^1^ for further details. Replacing $\cos \theta _z=\sin \alpha _s$ from Eq. (3) into Eqs. (21), (20) and finally into Eq. (18), we arrive at:

22 $$\begin{aligned} E_{\textrm{b}}(n,\varphi )&=\frac{G_{\textrm{on}}(n)}{\Omega }\int \limits ^{\omega _{\textrm{s}}}_{\omega _{\textrm{r}}}\cos \theta (n,\omega )\Theta \left[\cos \theta (n,\omega )\right] \\&\quad \times \left[a_0+a_1\exp \left( -\frac{k}{\cos \theta _z}\right) \right]\, \textrm{d}\omega . \end{aligned}$$

The integral given by Eq. (22) must be numerically evaluated. The variable of integration, the hour angle $\omega$, varies from the hour angle at sunrise, $\omega _{\textrm{r}}$, to the hour angle at sunset, $\omega _{\textrm{s}}$. Note that the hour angle $\omega$ is measured in radians. We will compute the integral using the Simpson’s rule. To proceed, we should specify the integral limits $\omega _{\textrm{r}}$ and $\omega _{\textrm{s}}$. For this purpose, we take the city Tehran with latitude $\varphi =35.69^\circ N$ and $n=81$ i.e.; 21 March (Nowruz or vernal equinox in not a leap year). It turns out: $\delta (81)=23.45^\circ \sin (360^\circ )=0$ and $G_{\textrm{on}}(81)=1375~\frac{W}{m^2}$. The sunrise and sunset hour angles are the roots of the equation:

23 $$\begin{aligned} \omega _{\textrm{s}}=\cos ^{-1}(-\tan \delta (81)\tan \varphi )=\cos ^{-1}(0)=90^\circ \end{aligned}$$

Assuming a negative angle, we have $\omega _{\textrm{r}}=-\omega _{\textrm{s}}=-90^\circ$. To evaluate the integrals, we need to specify the numerical values of the transmission coefficient parameters $a_0$, $a_1$, and *k*. These values depend on the climate type, and are given by^1^:

24 $$\begin{aligned} a_0=r_0a^{*}_0;~a_1=r_1a^{*}_1;~k=r_kk^{*}. \end{aligned}$$

where

25 $$\begin{aligned} a^{*}_0&=0.4237 -0.00821(6 - A)^2, \end{aligned}$$

26 $$\begin{aligned} a^{*}_1&=0.5055 + 0.00595(6.5 - A)^2, \end{aligned}$$

27 $$\begin{aligned} k^{*}&=0.2711 + 0.01858(2.5 - A)^2. \end{aligned}$$

Here, *A* represents the observer’s altitude in kilometers, and the correction factors $r_0$, $r_1$, and $r_k$ are dependent on the climate. For Tehran, which is a mid-altitude city with an altitude of $A=1.2\,\textrm{km}$, the climate-dependent correction factors are $r_0=0.97$, $r_1=0.99$, and $r_k=1.02$. Taking these factors into account, the transmission coefficient parameters become:

28 $$\begin{aligned} a_0=0.228;\quad a_1=0.666;\quad k=0.308. \end{aligned}$$

**Figure 5:**
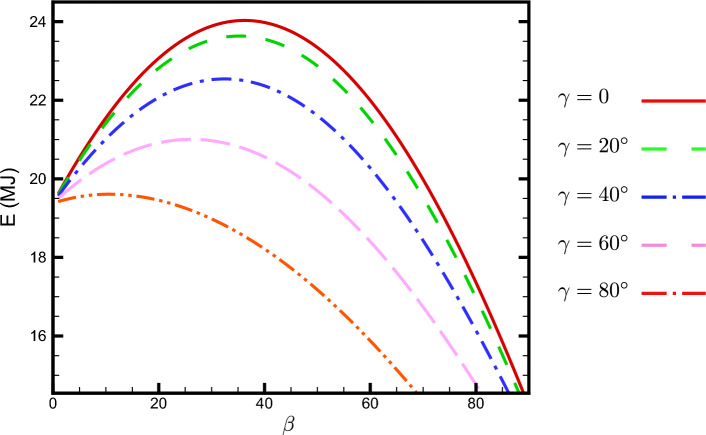
Direct beam irradiation energy $E_b$ (MJ) received on $n=81$ DoY by a flat plate of unit area versus its tilt angle $\beta$ for various values of azimuth angle $\gamma$. The plate is located at Tehran with latitude $\varphi =35.69^\circ N$.

Figure 5 shows the beam irradiation $E_b$ on a surface with unit area as a function of the tilt angle $\beta$, for various plane azimuthal angles $\gamma$, in Tehran on the day of Nowruz.

As shown in Fig. 5 for $\delta =0$ (corresponding to the day $n=81$), the beam irradiation energy received by a flat plate is maximized at the optimal angles $\gamma ^*=0$ and $\beta ^*=\varphi$. Let us prove this analytically. To obtain the beam irradiation energy received by a flat plate with arbitrary orientation $\beta$ and $\gamma$ (or $\eta$ and $\zeta$), one needs to evaluate the integral:

29 $$\begin{aligned} I_b=\int \limits ^{\omega _{\textrm{s}}}_{\omega _{\textrm{r}}}\cos \theta (n,\omega )\, \Theta [\cos \theta (n,\omega )]\, \tau _b\, \textrm{d}\omega . \end{aligned}$$

Note that $\sin \eta >0$ and it is independent of $\omega$. First, let us approximate the effective atmospheric optical transmission coefficient $\tau _b$ to be a constant. Then we should maximize the following integral

30 $$\begin{aligned} I_b(\eta , \zeta )= \tau _b \, \int \limits ^{\pi /2}_{-\pi /2}\cos \theta (n,\omega )\, \Theta [\cos \theta (n,\omega )]\textrm{d}\omega =: \sin \eta\ J(\zeta ). \end{aligned}$$

Here we have used Eq. (9). The Sun’s path is symmetric with respect to the plane normal to the equatorial plane passing through the zenith. Due to this symmetry, it is evident that $\zeta =0$ corresponds to the extremum value of the received energy, and hence $J(\zeta )$ is maximized. For any positive solution of $\zeta$ that leads to an optimal value for *E*, there should exist a negative solution as well. To complete the proof, let us show this directly. We may take $0\le \zeta \le \frac{\pi }{2}$ without any loss of generality. The same argument applies if we take $-\frac{\pi }{2}\le \zeta \le 0$.

31 $$\begin{aligned} J(\zeta )&=\tau _b \, \int \limits ^{\pi /2}_{-\pi /2}\cos (\omega -\zeta )\, \Theta [\cos (\omega -\zeta )\textrm{d}\omega \nonumber \\&=\dfrac{\tau _b }{2}\int \limits ^{\pi /2-\zeta }_{-\pi /2-\zeta }\left[ \cos (u)+|\cos (u)|\right] \, \textrm{d}u\nonumber \\&=\dfrac{\tau _b }{2}\int \limits ^{-\pi /2}_{-\pi /2-\zeta }\left[ \cos (u)+|\cos (u)|\right] \, \textrm{d}u+\dfrac{\tau _b }{2} \int \limits ^{\pi /2-\zeta }_{-\pi /2}\left[ \cos (u)+|\cos (u)|\right] \, \textrm{d}u\nonumber \\&=\tau _b \, \int \limits ^{\pi /2-\zeta }_{-\pi /2}\cos (u)\, \textrm{d}u =\tau _b \, (\cos \zeta +1). \end{aligned}$$

Note that we have used the identity: $x\Theta (x)=\frac{1}{2}(x+|x|)$ together with the change of variable $\omega -\zeta =u$. Maximizing the integral (30), we get:

32 $$\begin{aligned} \dfrac{\partial I}{\partial \eta }\big \vert _{\eta ^*, \zeta ^*}= & {} \cos \eta ^*\, J( \zeta ^*)=0, \end{aligned}$$

33 $$\begin{aligned} \dfrac{\partial I}{\partial \zeta }\big \vert _{\eta ^*, \zeta ^*}= & {} -\tau _b\sin \eta ^*\sin \zeta ^*=0, \end{aligned}$$

The first equation gives $\eta ^*=\frac{\pi }{2}$, and the second one leads to $\zeta ^*=0$. These are equivalent to $\gamma ^*=0$ and $\beta ^*=\varphi$. It is easily verified that $\frac{\partial ^2 I}{\partial \eta \partial \zeta }\big \vert _{\eta ^*,\zeta ^*}<0$ which proves that $\eta ^*=\frac{\pi }{2}$ and $\zeta ^*=0$ is a true maximum. Note that $J(\zeta )$ can be written as:

34 $$\begin{aligned} J(\zeta )=\tau _b\int \limits ^{\pi /2}_{-\pi /2}f(\omega ,\zeta )\, \textrm{d}\omega . \end{aligned}$$

where $f(\omega ,\zeta )=\cos (\omega -\zeta )\, \Theta [\cos (\omega -\zeta )$. Then $\frac{\textrm{d} J(\zeta )}{\textrm{d} \zeta }\big \vert _{\zeta =0}=0$ gives

35 $$\begin{aligned} \int \limits ^{\pi /2}_{-\pi /2}\dfrac{\partial f(\omega ,\zeta )}{\partial \zeta }\big \vert _{\zeta =0}\, \textrm{d}\omega =0. \end{aligned}$$

Here

36 $$\begin{aligned} \dfrac{\partial f(\omega ,\zeta )}{\partial \zeta }\big \vert _{\zeta =0}=\sin \omega \left( 1+\dfrac{\cos \omega }{|\cos \omega |} \right) , \qquad -\pi /2\le \omega \le \pi /2 \end{aligned}$$

which is an odd function with respect to $\omega$. In the realistic case where $\tau _b$ is not constant, $I(\eta ,\zeta )$ transforms to ${\tilde{I}}(\eta , \zeta )$ , where $\tau _b$ is a part of integrand. It turns out: ${\tilde{I}}(\eta ,\zeta )=\sin \eta \tilde{\,} J(\zeta )$ where

37 $$\begin{aligned} {\tilde{J}}(\zeta )&=\int \limits ^{\pi /2}_{-\pi /2}\cos (\omega -\zeta )\, \Theta [\cos (\omega -\zeta )]\,\tau _b(\varphi ,\omega )\textrm{d}\omega \nonumber \\&=\int \limits ^{\pi /2}_{-\pi /2}f(\omega ,\zeta )\,\tau _b(\varphi ,\omega )\textrm{d}\omega \end{aligned}$$

Maximizing with respect to $\eta$ still yields $\eta ^*=\frac{\pi }{2}$. One has

38 $$\begin{aligned} \dfrac{\textrm{d} {\tilde{J}}(\zeta )}{\textrm{d} \zeta }\big \vert _{\zeta =0}= \int \limits ^{\pi /2}_{-\pi /2}\dfrac{\partial f(\omega ,\zeta )}{\partial \zeta }\big \vert _{\zeta =0}\, \tau _b(\varphi ,\omega )\, \textrm{d}\omega \end{aligned}$$

**Figure 6:**
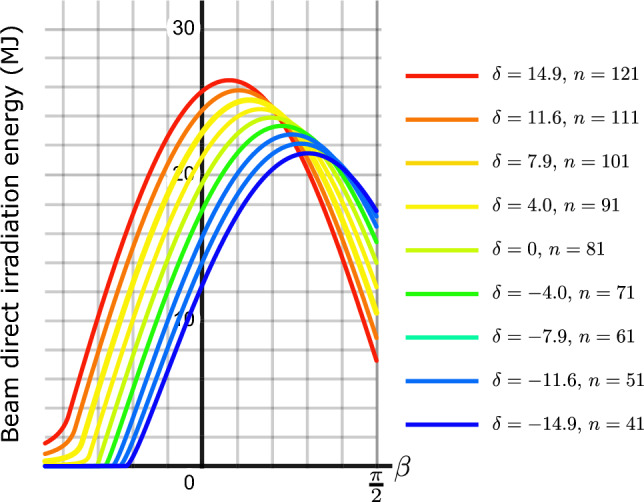
Beam direct irradiation energy (MJ) received by a flat plate of unit area on n-th DoY versus its tilt angle $\beta$ for various values of $\delta$. The plate is located at Tehran with latitude $\varphi =35.69^\circ N$. The plate’s azimuth angle is set to $\gamma =0$.

The integrand in the right-hand-side of (38) is the multiplication of two functions; an even function with respect to $\omega$, $\tau _b(\varphi ,\omega )$, and an odd function with respect to $\omega$. Then the integral vanishes. Therefore, $\zeta ^*=0$ and $\eta ^*= \dfrac{\pi }{2}$ (or equivalently $\gamma ^*=0$ and $\beta ^*=\varphi$) maximizes the received energy by the flat plate. For a general day of the year (DoY) represented by *n*, it can be shown, by a symmetry argument, that $\gamma ^*=0$ remains the optimal azimuth angle, but $\beta ^*$ depends crucially on *n*. Figure 6 shows the dependence of $E_b$ on $\beta$ for different values of *n*.

**Figure 7:**
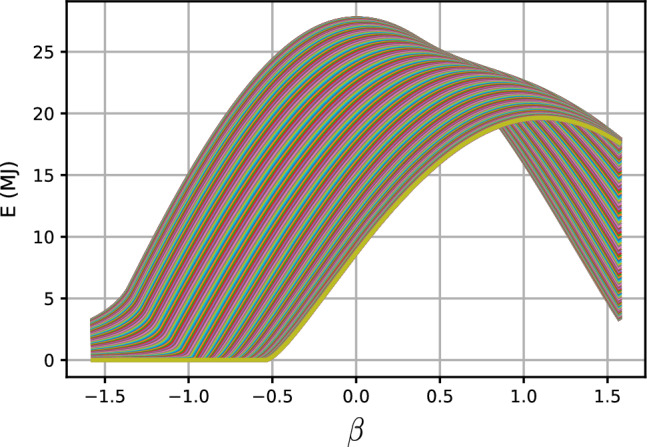
Daily beam direct irradiation energy (MJ) received by a flat plate of unit area as a function of its tilt angle $\beta$ (in radians). The lower (upper) curve corresponds to day of the year $n=354$ ($n=171$), representing the winter solstice and summer solstice, respectively. The plate is located in Tehran, Iran, with latitude $\varphi =35.69^\circ N$, and its azimuth angle is set to $\gamma =0$.

**Figure 8:**
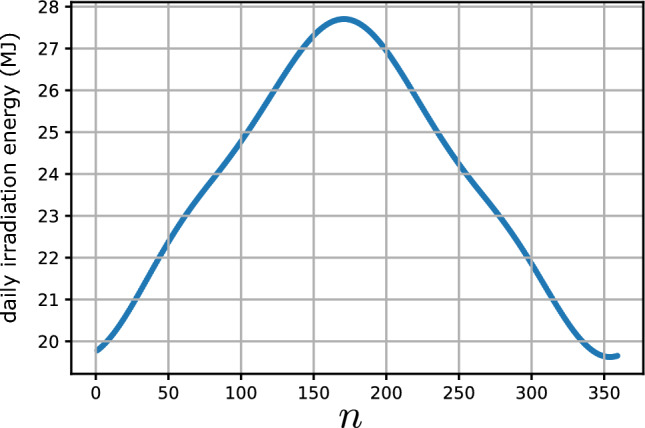
Maximum beam irradiation energy (MJ) received by an optimally oriented flat plate of unit area versus DoY, *n*. The plate is located at Tehran with latitude $\varphi =35.69^\circ N$ and its azimuth angle is set to $\gamma =0$. On the day $n=171$ the plate receives its annual maximal daily energy which is about 27.7 MJ.

**Figure 9:**
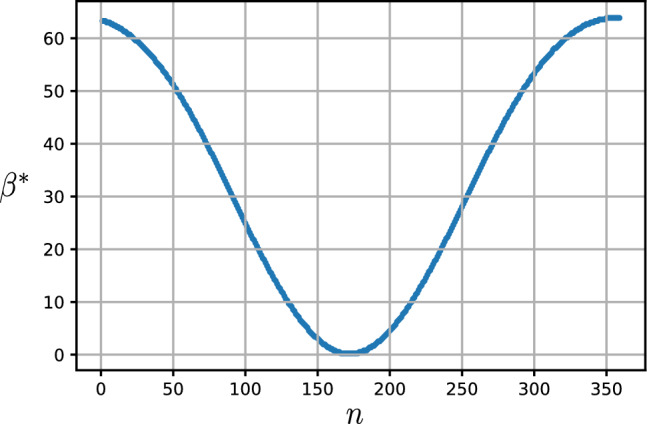
The optimum tilt angle $\beta ^*$ (degrees) versus DoY, *n*. The plate is located at Tehran with latitude $\varphi =35.69^\circ N$. The plate’s azimuth angle is set to $\gamma =0$.

Figure 7 shows the daily beam direct irradiation energy (in MJ) received by a flat plate of unit area as a function of its tilt angle $\beta$ for all days of the year $n=1,\cdots ,365$. Each graph in (7) represents the daily beam irradiation energy versus $\beta$ for a specific day of the year. The daily received energy, takes its maximum value at a day-dependent tilt angle $\beta ^*(n)$. Figure 8 shows the maximum beam irradiation energy (MJ) received by a flat plate of unit area located at Tehran with latitude $\varphi =35.69^\circ N$, the plate’s azimuth angle is set to zero. Figure 9 shows the optimal tilt angle $\beta ^*$ versus DoY, *n*.

Although we have presented data for a specific location (Tehran, Iran) in the Northern hemisphere, the qualitative behavior of the results remains the same for other locations with similar climate types.

### Sky diffuse contribution

Now we investigate the contribution of the sky diffusive irradiation component $E_d$ to the energy received by a tilted flat collector. There are different models that describe the directionality of diffusive irradiation. Here, we adopt the simpler assumption that the sky diffusive part of the solar irradiation is isotropic. The unit vector ${\varvec{e}}_r$, which represents the direction of the received diffusive radiation, can be specified by two angles: the polar angle $\vartheta$ and the azimuth angle $\phi$ in the local observer’s spherical system of coordinates. We have:

39 $$\begin{aligned} {\varvec{e}}_r={\varvec{k}}\sin \vartheta \sin \phi +{\varvec{e}}_{\textrm{s}}\sin \vartheta \cos \phi -{\varvec{e}}_{\textrm{w}}\cos \vartheta . \end{aligned}$$

**Figure 10:**
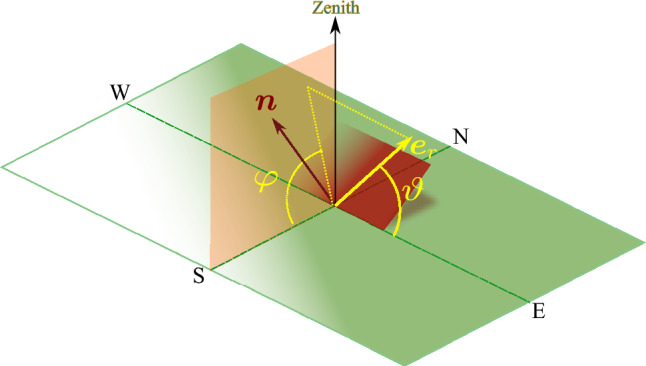
Incidence of sky diffusive radiation along the differential solid angle directed at $-{\varvec{e}}_r$ specified by two angles polar $\vartheta$ and azimuth $\varphi$ in the local observer’s spherical system of coordinates.

See Fig. 10 for illustration. As the symmetry argument still holds true in this case, we set $\gamma ^*=0$. Having the unit vector ${\varvec{n}}$ in Eq. (6) we find:

40 $$\begin{aligned} {\varvec{e}}_r\cdot {\varvec{n}}= \sin \vartheta \sin (\beta +\phi ). \end{aligned}$$

The contribution $E_d$ of the sky diffusive component to the received irradiated energy turns out to be

41 $$\begin{aligned} E_d(n,\omega )=\int \limits _{\mathbb {S}}\frac{G_d(n,\omega )}{\pi }(\textrm{d}\,\Omega )\,{\varvec{e}_r}\cdot {\varvec{n}}=\frac{G_d(n,\omega )}{\pi }I, \end{aligned}$$

where $G_d(n,\omega )$ is the sky diffusive irradiation on a horizontal surface of unit area and ${\mathbb {S}}$ is the angular integration region satisfying ${\varvec{e}_r}\cdot {\varvec{n}}\,\ge 0$. It turns out,

42 $$\begin{aligned} I&=\int \limits _0^{\pi -\beta } \textrm{d}\,\varphi \,\sin (\beta +\varphi )\int \limits _0^\pi \, \textrm{d}\,\vartheta \, \sin \vartheta \, \sin \vartheta ,\nonumber \\&=\frac{{\pi }}{2}\,(1+\cos \beta ). \end{aligned}$$

Diffuse irradiation appears to be isotropic as there is no preferred direction when we look at the sky. Many models have been proposed to study the contribution of diffuse irradiation. Interested readers may find a review on many of them in^47^. Here we adopt the version of the clear sky model proposed by Liu and Jordan in^48^. According to their model the instantaneous sky isotropic diffusive irradiation $G_d$ on a horizontal surface with unit area is given as follows:

43 $$\begin{aligned} G_d(n,\omega )=G_{\textrm{on}}(n)\tau _d(n,\omega )\cos \theta _z, \end{aligned}$$

where $\tau _d=0.271-0.294\tau _b$ is the atmospheric transmission coefficient of the diffusive irradiation. The instantaneous total diffuse irradiation on a tilted surface of unit area is given by:

44 $$\begin{aligned} G_{dT}(n,\omega )=\frac{1+\cos \beta }{2}G_{d}(n,\omega )=\frac{1+\cos \beta }{2}G_{\textrm{on}}(n)\tau _d\,\,\cos \theta _z. \end{aligned}$$

The daily energy received by a tilted flat plate due to the sky diffusive irradiation becomes:

45 $$\begin{aligned} E_{\textrm{d}}(n,\varphi )=\frac{1}{\Omega }\int \limits ^{\omega _{\textrm{s}}}_{\omega _{\textrm{r}}}G_{\textrm{dT}}(n,\omega )\ d\omega . \end{aligned}$$

Substituting $G_{dT}(n,\omega )$ from (44), the integral in (45) becomes:

46 $$\begin{aligned} E_{\textrm{d}}(n,\varphi )=\frac{1+\cos \beta }{2}\,\frac{G_{\textrm{on}}(n)}{\Omega }\int \limits ^{\omega _{\textrm{s}}}_{\omega _{\textrm{r}}}\tau _d\,\,\cos \theta _z\ d\omega . \end{aligned}$$

**Figure 11:**
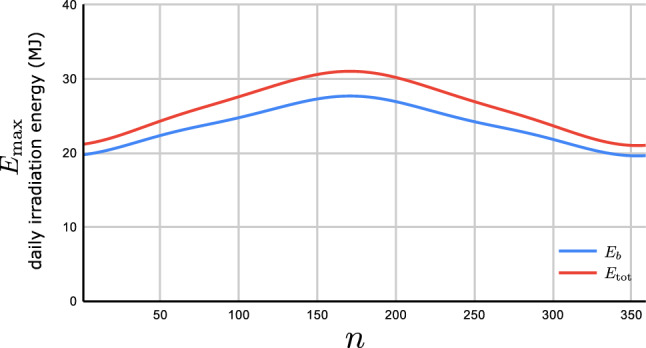
Daily maximum total irradiated energy $E_{\textrm{tot}}$ and beam irradiated energy $E_b$ (MJ) received by a flat plate of unit area versus *n*. The plate is located at Tehran with latitude $\varphi =35.69^\circ N$. The plate’s azimuth angle is set to the optimal value $\gamma ^*=0$.

This integral can be numerically evaluated for a given tilt angle $\beta$ and DoY. Our code is able to find the optimal value $\beta ^*$ for the total received energy $E_{\textrm{tot}}=E_b+E_d$. Figure 11 shows the maximal daily total energy $E_{\textrm{tot}}$ and beam component $E_b$ impinged on a flat plate with unit area. As it is seen the diffusive irradiation enhances the total received energy $E_{\textrm{tot}}=E_b+E_d$.

**Figure 12:**
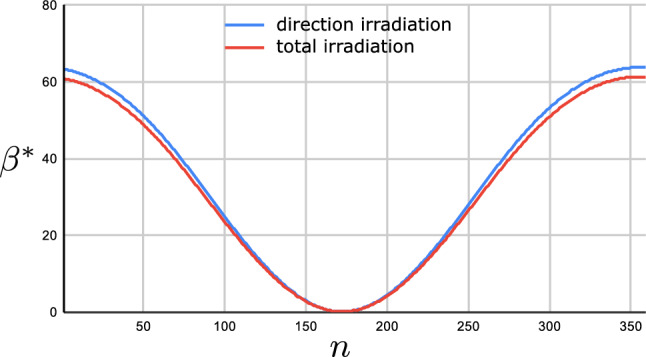
Plot of the optimum daily tilt angle $\beta ^*$ versus *n* for both $E_b$ and $E_{\textrm{tot}}$. The plate is located at Tehran with latitude $\varphi =35.69^\circ N$. The plate’s azimuth angle is set to $\gamma =0$.

Figure 12 shows the daily optimum tilt angle $\beta ^*$ both for the direct $E_b$ and the total $E_{\textrm{tot}}$ energies versus *n*. As you see, the optimal values are very close to each other.

## Comparison with a tracking flat plate

It is highly desirable to determine the amount of radiation energy that a flat collector would receive if it tracks the Sun. As the plate tracks the beam of the Sun, it maximizes its solar exposure by orienting perpendicularly to the beam. To answer this question, the received energy is divided into two parts: direct beam and diffuse sky radiation. Evaluating the contribution of direct beam energy is relatively straightforward. From a mathematical perspective, it is necessary to maintain the incidence angle $\theta$ at zero, which means that the unit normal vector ${\varvec{n}}$ is always along the solar vector ${\varvec{\sigma }}$ throughout the day. By equating Eqs. (1) and (6), we obtain $\beta =\frac{\pi }{2}-\alpha _s=\theta _z$ and $\gamma =\gamma _s$. For example, for an observer at the equator ($\varphi =0$) and on $n=81$, where $\delta =0$, we have according to (3) $\beta =\omega$. The total amount of direct beam energy $E^{\textrm{trc}}_b$ a tracking flat plate receiver can gain in the *n*th DoY can be obtained by setting $\theta =0~(\cos \theta =1)$ in integral (22). It turns out:

47 $$\begin{aligned} E^{\textrm{trc}}_{\textrm{b}}(n,\varphi )=\frac{G_{\textrm{on}}(n)}{\Omega }\int \limits ^{\omega _{\textrm{s}}}_{\omega _{\textrm{r}}}\left[ a_0+a_1\exp \left( -\frac{k}{\cos \theta _z}\right) \right] \, \textrm{d}\omega . \end{aligned}$$

The integral in (47) can numerically be computed. Next, the sky diffusive contribution is investigated. For a tracking plate, we can consider it as a fixed plate with an instantaneous tilt angle $\beta$ with $\cos \beta ={\varvec{k}}\cdot {\varvec{n}}={\varvec{k}}\cdot {\varvec{\sigma }}=\sin \alpha _s$. Replacing $\cos \beta$ with $\sin \alpha _s$ in Eq. (44) the instantaneous sky diffusive irradiation of a tracking flat plate becomes:

48 $$\begin{aligned} G^{\textrm{trc}}_{d}(n,\omega )=\frac{1+\sin \alpha _s}{2}\, G_{\textrm{on}}(n)\tau _d\,\,\cos \theta _z. \end{aligned}$$

The sky diffusive contribution to the daily received energy of a tracking plate can be obtained via the following integration:

49 $$\begin{aligned} E^{\textrm{trc}}_{\textrm{d}}(n,\varphi )&=\frac{1}{\Omega }\int \limits ^{\omega _{\textrm{s}}}_{\omega _{\textrm{r}}}G^{\textrm{trc}}_{\textrm{d}}(n,\omega )\textrm{d}\omega \nonumber \\ {}&=\frac{G_{\textrm{on}}(n)}{2\Omega }\int \limits ^{\omega _{\textrm{s}}}_{\omega _{\textrm{r}}}(1+\cos \theta _z)\tau _d\cos \theta _z \ \textrm{d}\omega . \end{aligned}$$

**Figure 13:**
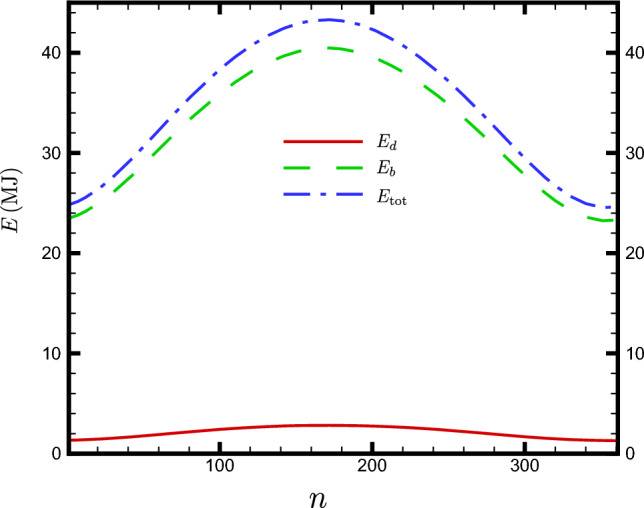
Dependence of separate sky diffusive $E_d$, beam $E_b$ and total $E_{\textrm{tot}}$ on *n* for a tracker flat plate of unit area. The plate is located at Tehran, Iran with latitude $\varphi =35.69^\circ N$.

Here, we have used the relationship $\sin \alpha _s=\cos \theta _z$. Figure 13 shows the daily dependence of the sky diffuse irradiance $E_d$, beam irradiance $E_b$, and the total received energy $E_{\textrm{tot}}$ for a flat plate tracker of unit area located in Tehran, Iran, with a latitude of $\varphi =35.69^\circ N$.

**Figure 14:**
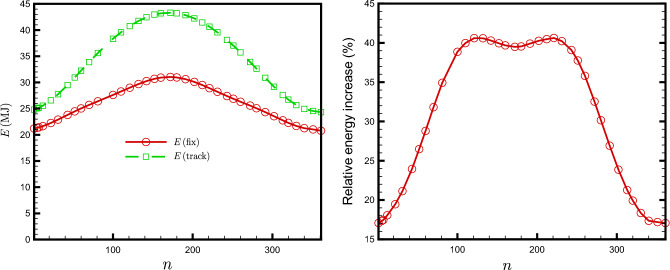
Daily dependence of the total energies, fix and tracking (left), and the relative energy increase percentage amount due to the tracking (right). The plate is located at Tehran, Iran with latitude $\varphi =35.69^\circ N$.

As expected, a tracking plate is able to capture more solar energy than a fixed plate with optimal orientation. Figure 14 illustrates the daily variation in the total energy received by both fixed and tracking plates, as well as the percentage increase in energy due to the tracking.

As you can see, the relative amount of the total energy increase percentage due to the Sun tracking critically depends on DoY. The minimum value of the relative energy increase percentage is about $17\%$ in early winter. It raises around $40\%$ in a relatively large interval starting around mid-May.

## Comparison to existing results in the literature

Most of the existing results are obtained for particular locations with different weather conditions. As far as we know, no similar results have been found for the city of Tehran. While some investigations have been conducted for cities in south of Iran near the Persian Gulf, none of them have taken clear sky conditions into account. The only papers that have taken the clear sky assumption into account are^14^, which investigated the city of Assiut in Egypt with latitude $\varphi =27.82^\circ N$, and^21^, which calculated the optimal tilt angles for representative days of each month at various latitudes on a daily basis. The qualitative dependence of both the daily-averaged optimal tilt angle and the maximal received energy at the daily optimal angle on the day of the year in Tehran and Assiut are similar to each other. This can be seen by comparing Figs. 11 and 12 with Fig. 3 of reference^14^. Furthermore, our results for the optimal tilt angle are in good agreement with those presented in^21^ for the latitude $\varphi =35^\circ N$, which is relatively close to the latitude of Tehran. To shed more light on the problem, we compared our findings with existing investigations that have taken into account weather conditions. Most of these papers have reported monthly averages at specific locations. To account for weather conditions, including cloudiness and pollutants, a clearness index ${\overline{K}}$ is typically introduced. This index represents the ratio of the monthly-averaged total energy received by a horizontal surface (direct, diffusive, and ground reflection) to the energy that a horizontal surface would receive outside of the atmosphere. It is defined as $K=\frac{{\overline{H}}}{\overline{H_{\textrm{o}}}}$, where ${\overline{H}}$ is the monthly-averaged received energy and $\overline{H_{\textrm{o}}}$ is the monthly average daily energy received outside the atmosphere. The monthly averaged total radiation energy received by a tilted surface of unit area on Earth is given by ${\overline{H}}_T=R{\overline{H}}$, where $R<1$ is a coefficient that can be estimated by considering the beam, diffuse, and reflected components of the radiation incidence on the tilted surface individually. Assuming that the diffuse and reflected radiation are isotropic, Liu and Jordan^48^ proposed that *R* can be expressed as:

50 $$\begin{aligned} R=(1-\overline{H_d}/{\overline{H}})R_b+\frac{\overline{H_d}}{{\overline{H}}}\frac{1+\cos \beta }{2}+\rho _g\frac{1-\cos \beta }{2}. \end{aligned}$$

Here, $\overline{H_d}$ is the average diffusive daily energy that a horizontal surface of unit area receives, $\rho _g$ is the ground albedo reflection coefficient, and $\beta$ is the surface tilt angle. Once $R_b$ and $\overline{H_d}$ are specified, the monthly average total energy that a tilted surface receives can be calculated. There are various models available for determining $\overline{H_d}$. Usually, people express $\overline{H_d}$ as a polynomial of the clearness index ${\overline{K}}$. Readers can refer to^27^ and^47^ for various models of $\overline{H_d}$ and the exact expression of $R_b$. The studies that are closest to the latitude of Tehran were conducted in^49,50^, which investigated eight cities in Turkey. Among them, Adana with a latitude of $\varphi =36.59^\circ N$ has the closest latitude to Tehran. For example, on March 16, the optimal tilt angles in Tehran (clear sky assumption) and Adana (realistic weather condition) are $33^\circ$ and $36^\circ$, respectively. Qualitatively speaking, the daily dependence of the optimal tilt angle is similar in both cities, despite the fact that the total energy is larger in Tehran than in Adana. In another study conducted for higher latitudes in the Northern hemisphere, the optimal tilt angle for Nottingham, England was found to be around $50^\circ$ in mid-March. In^21^, the optimal tilt angle for a latitude of $35^\circ$ was found to be $38^\circ$ in mid-March. Another study conducted for Abu Dhabi, which has a latitude lower than Tehran, gives the average optimal tilt angle for March at $25^\circ$. Naively speaking, the optimal tilt angle tends to decrease with decreasing latitude.

## Summary and conclusion

To summarize, we have analytically calculated the optimal tilt and azimuth angles for a fixed flat-type collector at a given latitude. The contribution of the ground-reflected solar radiation is neglected. We have separately evaluated the maximal energy received by a flat plate of unit area associated with the direct beam and sky diffuse components of irradiation. In a geocentric system of coordinates, it has been analytically shown that the optimal azimuthal angle of a collector is $\gamma ^*=0$, based on the Sun’s symmetrical motion with respect to the equatorial plane. However, the optimal tilt angle $\beta ^*$ crucially depends on the day of the year (*n*) and the local latitude. Analytical analysis has been performed for a mid-altitude climate location in Tehran, Iran. The maximal daily received energies, namely direct beam and sky diffuse, associated with this optimal orientation are compared to their corresponding values when the flat plate tracks the Sun. The relative amount of the total energy increase due to Sun tracking drastically depends on the day of the year. In early winter, the increase percentage is minimum and around $17\%$. In a relatively large interval starting from mid-May, the increase percentage is large, up to $40\%$. Although our results are associated with a particular location (Tehran) with a mid-altitude climate, the qualitative behavior for other latitudes remains the same. We have not found such a detailed comparison elsewhere in the literature.

## Data Availability

All data generated or analyzed during this study are included in this published article.
